# Preface

**DOI:** 10.1098/rsta.2012.0516

**Published:** 2013-07-13

**Authors:** Nora de Leeuw

**Affiliations:** University College London, London, UK

**Keywords:** surface science, interstellar medium

Chemical and physical processes occurring at the surfaces of the minute grains found in interstellar dust clouds are crucial in the formation of the many different atomic and molecular species found in the interstellar medium. These surface processes include not only relatively simple processes, such as the formation of dihydrogen gas from the atomic hydrogen prevalent in the interstellar medium, but also the assembly of small molecules, such as water and carbon dioxide and their catalysis into bigger, more complex species. When the dust grains coalesce into planetary accretion discs, these molecules may be retained and become trapped in the planets as they form. Direct measurements, laboratory studies, theoretical investigations and mathematical models all serve to improve our understanding of these complex processes.

This Theme Issue is the outcome of a Royal Society International Scientific Seminar on ‘Surface science in the interstellar medium’, which brought together an interdisciplinary group of scientists to explore how their complementary expertise in chemistry, physics, astronomy, computing and mathematics could be exploited to provide a fuller picture of this fascinating area of research. The papers presented here showcase a wide variety of state-of-the-art techniques used to shed light on the formation of the interstellar dust clouds and the surface-mediated reactions that convert simple atoms into increasingly complex molecules. They illustrate how this combination of expertise can unravel a range of complex physico-chemical processes occurring under the extreme conditions prevalent in the interstellar medium.

I would like to dedicate this Theme Issue to the memory of Prof. Michael J. Drake, a friend and colleague who was one of the driving forces behind the organization of the Royal Society meeting, but tragically died shortly afterwards. Mike had a profound interest in surface processes occurring in the interstellar medium. Having spent half a life-time teaching his students the accepted versions of the origin of our planetary water, which increasingly did not fit the available evidence, Mike suggested an alternative hypothesis, where water was already present at the surfaces of interstellar dust grains when they accreted to form our planet. Although this hypothesis fitted with all available evidence, it would only work if the adsorption of water to the dust grains was sufficiently strong to survive the harsh conditions in the accretion disc. Mike then had the insight to look outside his own field and to computer simulations to test his hypothesis. Simulations of water adsorption at dust grain models have shown that the kind of highly fractal surfaces found on interstellar dust grains are indeed suitable for the strong retention of water under the extreme temperatures and pressure conditions prevalent in the accretion disc during planetary formation. Some of this work is illustrated in this Theme Issue, which I trust is a fitting memorial to Mike's contributions to the field.

